# Are ultra-short heart rate variability features good surrogates of short-term ones? State-of-the-art review and recommendations

**DOI:** 10.1049/htl.2017.0090

**Published:** 2018-03-14

**Authors:** Leandro Pecchia, Rossana Castaldo, Luis Montesinos, Paolo Melillo

**Affiliations:** 1 School of Engineering, University of Warwick, Coventry, CV4 7AL, UK; 2 The Multidisciplinary Department of Medical, Surgical and Dental Sciences of the Second University of Naples, Naples, 80131, Italy

**Keywords:** statistical analysis, medical signal processing, electrocardiography, patient monitoring, ultrashort heart rate variability features, short-term heart rate variability features, HRV, healthcare applications, health monitoring, wearable sensors, mobile phones, smart watches, statistical tests

## Abstract

Ultra-short heart rate variability (HRV) analysis refers to the study of HRV features in excerpts of length <5 min. Ultra-short HRV is widely growing in many healthcare applications for monitoring individual's health and well-being status, especially in combination with wearable sensors, mobile phones, and smart-watches. Long-term (nominally 24 h) and short-term (nominally 5 min) HRV features have been widely investigated, physiologically justified and clear guidelines for analysing HRV in 5 min or 24 h are available. Conversely, the reliability of ultra-short HRV features remains unclear and many investigations have adopted ultra-short HRV analysis without questioning its validity. This is partially due to the lack of accepted algorithms guiding investigators to systematically assess ultra-short HRV reliability. This Letter critically reviewed the existing literature, aiming to identify the most suitable algorithms, and harmonise them to suggest a standard protocol that scholars may use as a reference in future studies. The results of the literature review were surprising, because, among the 29 reviewed papers, only one paper used a rigorous method, whereas the others employed methods that were partially or completely unreliable due to the incorrect use of statistical tests. This Letter provides recommendations on how to assess ultra-short HRV features reliably and proposes an inclusive algorithm that summarises the state-of-the-art knowledge in this area.

## 1 Introduction

The dynamic modulation of heart rate (HR) is controlled by the several voluntary and involuntary mechanisms, including respiration, thermoregulation and the interaction of the sympathetic (which has a response time in the order of a few seconds) and parasympathetic activities (which works much faster: response time 0.2–0.6 s) [1]. Those modulations result in HR fluctuation or variability in time. Whereas the measure of HR is a static index of autonomic input to the sinus node, which does not provide direct information on sympathetic or parasympathetic functions, HR variability (HRV) analysis provides a quantitative assessment of cardiac autonomic regulation [2].

According to [3], HRV refers to the time series of the interval variation between consecutive heart beats and it can be analysed in time, frequency and non-linear domains [3, 4]. Common HRV features extracted from HRV excerpts are reported in Table 1.

**Table 1:** HRV features

HRV measures	Units	Description
**time domain**		
MeanNN	[ms]	mean of NN intervals
StdNN	[ms]	standard deviation of NN intervals
MeanHR	[1/min]	mean HR
StdHR	[1/min]	standard deviation of instantaneous HR values
RMSSD	[ms]	square root of the mean squared differences between successive NN intervals
NN50	—	number of successive NN interval pairs that differ more than 50 ms
pNN50	[%]	NN50 divided by the total number of NN intervals
HRV triangular index	—	integral of the NN interval histogram divided by the height of the histogram
TINN	—	baseline width of the NN interval histogram
**frequency domain**		
VLF	[ms^2^]	VLF power (0.0033–0.04 Hz)
LF	[ms^2^]	LF power (0.04–0.15 Hz)
HF	[ms^2^]	HF power (0.15–0.4 Hz)
LF_peak_, HF_peak_	[Hz]	LF and HF band peak frequency
LFnu, HFnu	Nu	LF and HF power normalised
LF/HF	—	Ratio of LF and HF band powers
TotPow	[ms^2^]	total power
**non-linear domain**		
SD1, SD2	[ms]	standard deviation of the Poincare’ plot perpendicular to (SD1) and along (SD2) the line-of-identity
ApEn	—	approximate entropy
SampEn	—	sample entropy
D2	—	correlation dimension
dfa1, dfa2	—	detrended fluctuation analysis: short-term and long-term fluctuation slope
RPlmean	[beats]	recurrence plot analysis: mean line length
RPlmax	[beats]	recurrence plot analysis: maximum line length
REC	[%]	recurrence rate
RPadet	[%]	recurrence plot analysis: determinism
ShanEn	—	Shannon entropy

HRV analysis can be performed on 24 h nominal recordings (referred as long-term HRV analysis), 5 min recordings (referred as short-term HRV analysis) or shorter recordings [3], which in this review is referred as ultra-short term HRV analysis. Since clear guidelines on ultra-short HRV analysis are not available yet, this review aimed to explore to what extent ultra-short HRV features can be used to estimate short-term ones, which are still to be considered as a benchmark for HRV analysis. In medicine, and particularly in clinical trial design, in order to cope with this kind of problem, the concept of a surrogate endpoint (or marker) was introduced [5, 6]. A surrogate measure is a marker, which is used to estimate a real clinical endpoint, when this is undesired (e.g. death) or when it cannot be directly observed or measured. Several regulatory bodies (e.g. FDA and NICE) have started to accept evidence from clinical trials that show a direct clinical benefit in using surrogate markers. Proving whether a marker is a good surrogate of a real clinical outcome can be quite difficult, and a combination of appropriate statistical and correlation tests is required. Although a rich literature has been produced to answer this question, still some authors demonstrated to be confused and the sentence ‘*a correlate does not make a surrogate*’, first used by Fleming *et al.* [5], became a mantra in this field. In fact, there is a common misconception that if a marker correlates with the true clinical outcome, it can be a valid surrogate endpoint, replacing the true clinical outcome. However, a much stronger condition than correlation is required to be sure that a surrogate is valid and can be used to replace a real clinical outcome. Another common misconception is that a marker X can be considered a good surrogate of a clinical outcome Y if statistical null-hypothesis tests demonstrate no-significant differences between X and Y. This is a major misconception because statistical differences may reveal themselves only in particular conditions (e.g. when a sufficient number of measures are observed). In addition, both correlation and statistical tests are often used improperly (e.g. parametric tests used for non-normally distributed features).

From the theoretical point of view, it should be well-known that some HRV features lose significance if computed in ultra-short term [3]. For instance, it is recommended that spectral analyses are performed on stationary recordings lasting at least 10 times more than the slower significant signal oscillation period. In the case of short-term HRV analysis, the slower significant oscillations in the so-called low-frequency (LF) power spectrum bandwidth have a period of 25 s (i.e. frequency of 0.04 Hz). Thus, in order to measure the entire LF power spectrum of HRV excerpts (i.e. including the slower components) at least 250 s of HRV signals are required. In the same manner, in order to compute the high-frequency (HF) power, at least 1 min is required [3]. Therefore, LF and HF power spectra computed in excerpts shorter than 1 min lead to erroneous results. As far as non-linear HRV features, less has been explored in the existing literature. Moreover, approximate entropy (ApEn) has shown to be unreliable in excerpts lasting <3 min [7].

The demands of ultra-short term HRV analysis for monitoring individual's health and well-being status in real life is significantly increasing, especially in relation to wearable sensors or mobile applications. Out of the lab, in fact, the conventional 5 min HRV recordings might be unsuitable, due to the real-time requirements. In fact, ultra-short recording may allow continuous and quasi-real-time monitoring of an individual's well-being status (i.e. mood, attention, and stress levels) [8]. Many apps and wearable devices are being released into the market, claiming to do HRV analysis in real time (from 10 s to 1 min). Although there is a clear need for such technologies, unfortunately, two problems remain unsolved:

- there are not yet clear guidelines on how to analyse HRV in the ultra-short term and which ultra-short HRV features can be considered as good surrogates of short-term ones;
- there is no clear algorithm to identify reliable ultra-short HRV features for the detection of an event.

In analogy with evidence-based medicine, this Letter provides a critical review of the state-of-the-art methods used to assess ultra-short HRV validity, providing key recommendations on how to assess ultra-short HRV features that are good surrogates of short-term ones. As described by Grant *et al.* [9], there are different typologies of literature reviews. According to our previous experiences [8, 10–12], the typology of the review is strongly depended on the heterogeneity and the quality of the available published literature, which in this case did not allow a more mathematical pooling (e.g. a meta-analysis). Nonetheless, several authors gave effective methodological contributions, although in a fragmented manner. Consequently, this Letter aimed to harmonise these contributions in a comprehensive algorithm that can be useful to guide scholars in future studies.

## 2 Methods and materials

Relevant studies on the use of ultra-short HRV analysis were first identified and selected by searching on PubMed and OvidSP databases. Articles were searched using Boolean combinations of the following keywords or their equivalent medical subject heading terms: heart rate variability, HRV, ultra-short, and very short. Title, abstract, and full text were chosen as fields of the search. However, due to the lack of guidelines on how to analyse HRV in the ultra-short term, the nomenclature used in many scientific papers was very heterogeneous, if not misleading. For instance, many studies performing HRV analysis on segments <5 min did not use the tag ‘ultra-short term’ or did not mention the length of HRV excerpts analysed (i.e. ultra-short, short- or long-term analysis) in the study. Therefore, a linear search of references of retrieved articles was required and performed.

The heterogeneity and quality of available literature led us to conduct a state-of-art review to address the current concerns and offer a comprehensive perspective on the issue [9].

To limit the linear search, the following criteria were utilised: papers published in the last 15 years (since 2003), focusing on healthy and non-pregnant adult humans. Shortlisted papers were considered suitable for this review if they met the following criteria:

- the subjects were human beings over 18 years old;
- HRV was analysed on excerpts <5 min;
- HRV features were extracted with reliable methods and reported with sufficient statistical quality [3].

## 3 Results and discussion

**Fig. 1:**
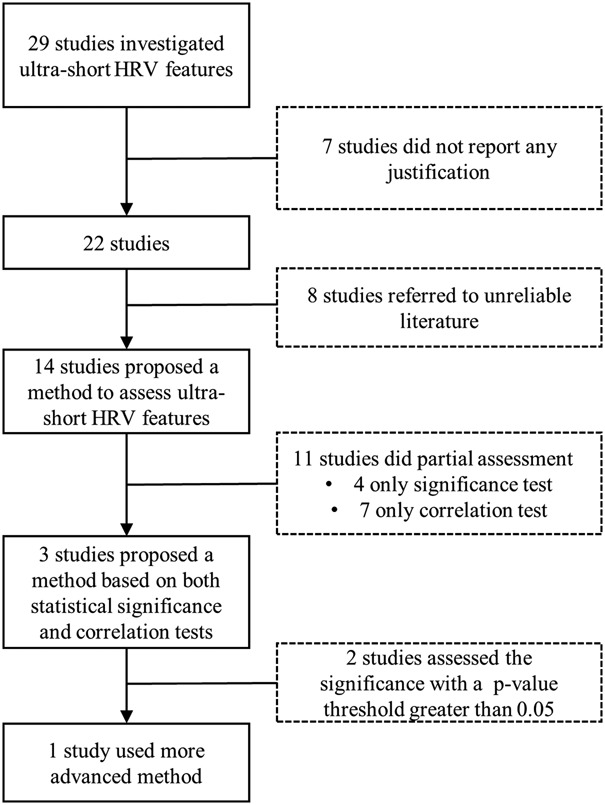
Flow chart of the literature search

Since 2003, 29 papers [13–41] were identified as shown in Fig. 1. An overview of the methods employed in the shortlisted 29 papers to assess the validity of ultra-short HRV features is synthetically reported in Fig. 1, whereas the characteristics of the reviewed studies are reported in Table 2.

**Table 2:** Characteristics of studies

Author, year	HRV features investigated	Length, s	Condition	N. Sub	Justification for ultra-short HRV adoption
Arza, 2015 [40]	MeanHR, StdNN, RMSSD, pNN50, VLF, LF, HF, LF/HF and LFnu	180	rest/stress	25	• none
Baek, 2015 [37]	MeanHR, StdNN, RMSSD, pNN50, VLF, LF, HF, LF/HF, TotPow, LFnu, HFnu	270–10	control	500	• Stat.: Kruskal–Wallis test (*p* > 0.05) • Cor.: Pearson's correlation analysis and Bland–Altman plot
Boonnithi, 2011 [14]	MeanNN, StdNN, MeanHR, StdHR, RMSSD, pNN50, VLF, LF, HF, LF/HF, LFnu, HFnu	50	rest/stress	6	• referred to the literature [20]
Brisinda, 2014 [15]	All features reported in Table 1, except HRV index and TINN	120, 60, 30	rest/stress	113	• Cor.: ICC
Choi, 2009 [26]	LF, HF, LF/HF	240	rest/stress	3	• none
De Rivecourt, 2008 [25]	MeanHR, LF and HF	240, 120, 60, 30	rest/mental workload	19	• Cor.: Pearson's on log transformed features
Esco, 2014 [30]	RMSSD	60, 30, 10	pre/post exercise	23	• Stat.: ANOVA (*p* > 0.05), Cohen's *d* • Cor.: ICC and Bland–Altman graph on log transformed features
Flatt, 2013 [34]	RMSSD	55	control	25	• referred to the literature [20, 35]
Hjortskov, 2004 [27]	LF, HF and LF/HF	180	rest/stress	12	• none
Kim, 2008 [19]	StdNN, RMSSD, pNN50, HRV index, TINN, LF, HF	180	rest/stress	68	• referred to the literature [20, 41]
Kwon, 2016 [39]	StdNN, RMSSD, MeanHR, LF, HF, LF/HF, TotPow, LFnu and HFnu	30	control	14	• referred to the literature [20]
Li, 2009 [29]	MeanNN, RMSSD and HF	30	rest/stress	399	• Cor.: Pearson on log transformed features
Mayya, 2015 [16]	StdNN, RMSSD, pNN50, LF, HF, LF/HF, SD1, SD2, and dfa1	60	rest/stress	49	• referred to the literature [20]
McNames, 2006 [36]	MeanHR, StdNN, RMSSD, LF, HF, LF/HF, TotPow, LFnu and HFnu	600–10	control	54	• Cor.: ICC
Munoz, 2015 [33]	StdNN and RMSSD	120, 30, 10	control	3.387	• Cor.: Pearson and Bland–Altman plot on log transformed features • Stat.: Cohen's *d*
Nardelli, 2017 [31]	SD1 and SD2	60, 25, 15	rest/sound	32	• Cor.: Spearman correlation and Bland–Altman plot
Nussinovitch, 2011 [35]	MeanNN, StdNN, RMSSD, HRV index, pNN50, LF, HF, TotPow	60–10	control	7	• Cor.: ICC
Pandey, 2016 [17]	MeanNN, StdNN, MeanHR, StdHR, RMSSD, VLF, LF and HF	60	rest/stress	15	• none
Papousek, 2010 [22]	MeanHR, LF, HF and LF/HF	180	rest/stress	65	• none
Pereira, 2017 [21]	MeanNN, StdNN, RMSSD, pNN20, pNN50, LF, HF, LF/HF, LFnu, SD1, SD2, SampEn and dfa1	220–50	rest/stress	14	• Stat.: ANOVA between rest and stress at different time scale (*p* < 0.05)
Salahuddin, 2007 [20]	MeanNN, RMSSD, pNN50, HRV index, TINN, VLF, LF, HF, LF/HF, LFnu, and HFnu	150–10	rest/stress	24	• Stat.: Kruskal–Wallis test at each condition between 5 min and each time length (*p* > 0.05), and Wilcoxon sign-ranked test between rest and stress at different time length (*p* < 0.05)
Salahuddin, 2007 [41]	MeanNN, RMSSD, pNN50, HRV index, TINN, VLF, LF, HF, LF/HF, LFnu, and HFnu	150–10	control	6	• Stat.: Kruskal–Wallis test (*p* > 0.05)
Schroeder, 2004 [38]	MeanNN, StdNN, MeanHR, RMSSD, HF, LF, LFnu, HFnu	360, 180, 10	control	63	• Cor.: ICC on log transformed features, and multivariate repeated measures
Schubert, 2009 [24]	MeanHR, StdNN, LF, HF, LF/HF and D2	180	rest/stress	50	• none
Sun, 2010 [23]	MeanNN, StdNN, MeanHR, StdHR, RMSSD, pNN50, LF, HF, LF/HF	60	rest/stress	20	• referred to the literature [20]
Thong, 2003 [32]	SDNN, RMSSD and HF	300–10	control	25	• Stat.: two-way ANOVA (*p* > 0.05),
Wang, 2009 [28]	MeanNN, RMSSD and HF	30	rest/stress	735	• referred to the literature [25, 38]
Wijsman, 2011 [18]	MeanHR, StdNN, LF, HF and LF/HF	120	rest/stress	30	• none
Xu, 2015 [13]	MeanHR, pNN50, LF, HF, LF/HF	180, 30	rest/stress	44	• referred to the literature [20]

ICC: inter-class correlation analysis.

The studies focused on ultra-short HRV features for different purposes: 18 focused on mental stress or mental workload detection [13–29]; one focused on athletic performance monitoring [30]; one focused on auditory stimuli [31]; the remaining investigated the reliability of ultra-short HRV features in control condition (e.g. only resting condition). The 18 studies investigating mental stress used one or more of the following tasks to induce mental stress: computer work task, flight simulator, Stroop colour word task, arithmetic task, memory task, logic task, game task, public speech task, academic examination, and other physical–mental tasks. Ten papers [25–34] investigated three or fewer HRV features. De Rivecourt *et al.* [25] explored MeanHR, LF, and HF as indices for momentary changes in the mental effort during simulated flight. However, only two HRV frequency features were investigated at different lengths (i.e. 240, 120, 60 and 30 s). Choi *et al.* [26] investigated frequency HRV features to detect stress using 240 s excerpts. Hjortskov *et al.* [27] explored only three HRV frequency features (LF, HF and LF/HF) in 3 min segments during rest and computer working. Wang *et al.* [28] investigated MeanNN, RMSSD, and HF during rest and stress sessions at 30 s. Li *et al.* [29] investigated MeanNN, RMSSD, and HF in 30 s compared with all the durations of rest and stress sessions (i.e. 10 min). Esco *et al.* [30] only investigated RMSSD during pre and post exercise, as RMSSD showed to be a reliable feature to assess performances in athletes. They investigated RMSSD at different time scales of 10, 30, and 60 s. Nardelli *et al.* [31] investigated Poincare’ plot features (SD1 and SD2) at 15, 25 and 60 s during a control condition and effective sound. Thong *et al.* [32] investigated three HRV features StdNN, RMSSD, and HF at different time scales from 10 to 300 s (with a step of 10 s) at control condition. Munoz *et al.* [33] investigated only two HRV features: StdNN and RMSSD at 10, 30 and 120 s during a control condition. Flatt *et al.* [34] investigated one HRV feature, RMSSD, at 55 s during a control condition. The remaining studies investigated more than three HRV features.

As shown in Fig. 1, seven out of the 29 studies [17, 18, 22, 24, 26, 27, 40] did not report any method to validate the use of ultra-short HRV features or reference to support the adoption of ultra-short HRV features. Eight studies [13, 14, 16, 19, 23, 28, 34, 39] also did not report any method to validate the use of ultra-short HRV features but they relied on the results of five previous studies [20, 25, 35, 38, 41]. Unfortunately, those five studies are part of 11 studies that cannot be considered fully reliable as detailed below.

In fact, 11 identified studies [15, 20, 21, 25, 29, 31, 32, 35, 36, 38, 41], including the five mentioned in the previous sentence [20, 25, 35, 38, 41], performed only a partial assessment either using only statistical significance or performing only correlation tests. In fact, three of 11 studies [20, 32, 41] employed statistical significance tests to prove that there were no statistically significant changes in HRV features in short versus ultra-short term, assuming short-term HRV analysis (i.e. 5 min) as a benchmark. They concluded that ultra-short HRV features were good surrogates of short-term ones if no-significant differences were observed, using a significance threshold >0.05 (*p* > 0.05).

Unfortunately, this result is arguable because, although a *p*-value <0.05 is conventionally used to support the hypothesis that two distributions are significantly different, it is well-known that no conclusions can be drawn for *p*-value >0.05, as detailed in [42]. For instance, two distributions could result in a *p*-value >0.05 because of their cardinalities. In particular, one of those three studies [20] also assessed ultra-short term HRV features in two conditions (i.e. rest and stress) using a non-parametric test (*p* < 0.05) to find the shortest duration needed to distinguish the two conditions. Nevertheless, also, in this case, the results are arguable as the study [20] explored only those HRV features judged as good surrogates if no statistically significant changes in short versus ultra-short term were observed using a *p*-value >0.05. Furthermore, one study [21] used one-way analysis of variance (ANOVA) to determine which HRV features (i.e. those computed at 220, 150, 100 or 50 s) could discriminate between rest and stress sessions with *p* < 0.05. However, due to the nature of HRV features, which are non-normally distributed (especially in the frequency domain), a non-parametric test should have been used instead, or HRV features should have been log-transformed before using the ANOVA test.

On the other side, seven studies [15, 25, 29, 31, 35, 36, 38] employed only correlation tests to prove that ultra-short term HRV features behaved as short-term ones; in fact, they concluded that ultra-short HRV features were good surrogates of short-term ones if significantly correlated with their equivalent short HRV features. As anticipated in the introduction, this result is arguable because as stated by Fleming *et al.* [5], ‘a correlate does not make a surrogate’, although an appropriate correlation test is the first step for the identification of a good surrogate.

Only two studies [30, 37] performed both statistical significance test and correlation analysis. Unfortunately, also in these two studies, the statistical significance analysis consisted of only observing if the *p*-value was >0.05, which is not a suitable method for the reasons discussed above.

Employing invalid statistical significance analysis led to unreliable results, especially regarding frequency HRV features. In fact, Baek *et al.* [37] and Salahuddin *et al.* [41] computed very low frequency (VLF) in 270 and 50 s although, as reporting also in [3], VLF is only reliable in long-term HRV analysis. De Rivecourt *et al.* [25] and Salahuddin *et al.* [41] employed only correlation analysis and an inaccurate statistical significance test (i.e., *p* > 0.05), reported that LF and HF are reliable in segments lower than 30 s, whilst at least 250 and 60 s are necessary for LF and HF, respectively [3].

Finally, only one study [33] investigated in a more rigorous way the validity of ultra-short HRV features. In fact, Munoz *et al.* [33] compared 10, 30, and 120 s HRV features with 5 min ones, using Pearson's correlation test (after having normalised HRV features with log-transformation), Bland–Altman plots and Cohen's *d* statistical test. Unfortunately, Munoz *et al*. reported the results on only two time domain HRV features under one condition (i.e. resting) and it was not clear if other features were computed but not reported or not computed at all. In the first case, a correction to the *p*-value should be employed too [43, 44].

Hence, among the 29 identified papers, one paper justified the adoption of ultra-short HRV features with a rigorous method but reporting only on two time domain HRV features. Conversely, seven papers did not provide any justification, eight papers based their choice on unreliable articles, 11 papers performed only a partial assessment (i.e. either statistical significance or correlation tests) and two papers performed a complete assessment (both statistical significance and correlation tests) but using statistical significance tests improperly. Overall, none of the 29 studies has proposed a valid method to identify reliable subsets of ultra-short HRV features or surrogates of the short-term HRV features to allow the detection of the event of interest (i.e. two different conditions). Therefore, future studies in this area are required.

Independent of the methods used (e.g. statistical test and correlation, only statistical or correlation analysis) and their rigor (e.g. the parametric test used for non-normally distributed features, *p* > 0.05), the reviewed studies presented other methodological ambiguities. Twenty studies investigating ultra-short HRV analysis in two conditions (e.g. rest versus stress), compared ultra-short HRV features inter-group (e.g. ‘HRV features at 1 min during rest versus stress’ compared with ‘HRV features at 5 min during rest versus stress’) without performing intra-group (e.g. ‘HRV 1 min at rest’ versus ‘HRV 5 min at rest’) comparisons. In fact, inter-group (i.e. comparing HRV features between two conditions among different lengths) and intra-group comparisons (i.e. comparing coherence of HRV features at different lengths in the same condition) should be performed using the proper statistical tests and correlation analyses. This is fundamental in order to judge the inner validity of the technique.

**Fig. 2:**
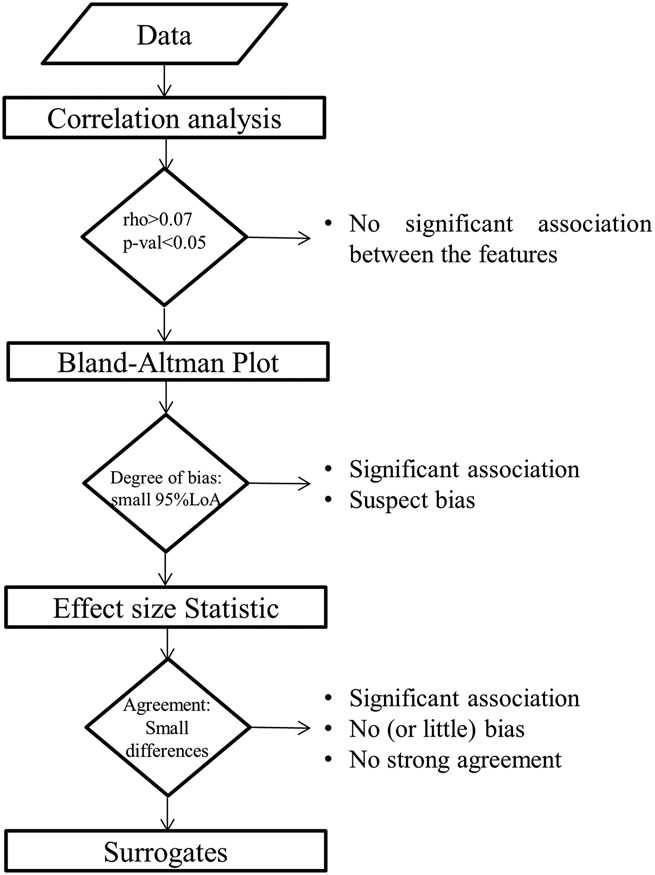
Standard algorithm to assess if ultra-short HRV features can be considered good surrogate for short-term ones when investigating one condition (e.g. only at rest). rho: correlation coefficient; p-val: p-value associated with correlation analysis; LoA: line of agreement in Bland–Altman plot

Overall, the reviewed literature highlighted that some valuable methodologies are available and already in use, but in a very fragmented way, resulting in improper or inaccurate practices. This body of evidence can be summarised and standardised in an algorithm, as represented in Fig. 2.

The algorithm represented in Fig. 2 highlights that authors cannot just use statistical or correlation tests to explore whether ultra-short HRV features can be considered good surrogates of short-term ones. Before performing statistical tests, authors should consider if features are significantly correlated at different time scales. The significant correlation suggests that there is a significant association. Nonetheless, this association could be biased. The Bland–Altman estimates this bias and how it diverges with the increase of the short-term feature's magnitude (i.e. benchmark). According to this test, two features are considered not biased, if the dispersion of their mean difference remains within a conventional threshold [i.e. 95% line of agreement (LoA)] [45]. Once a correlation has been proven and bias excluded, the statistical significance can be explored. Munoz *et al.* [33] proposed the use of the Cohen's *d* statistics to quantify the agreement of HRV features at different time scales relative to their within-group variation [46]. Therefore, according to the proposed algorithm, a feature can be considered a good surrogate if correlated, non-biased and significantly in agreement among them.

**Fig. 3:**
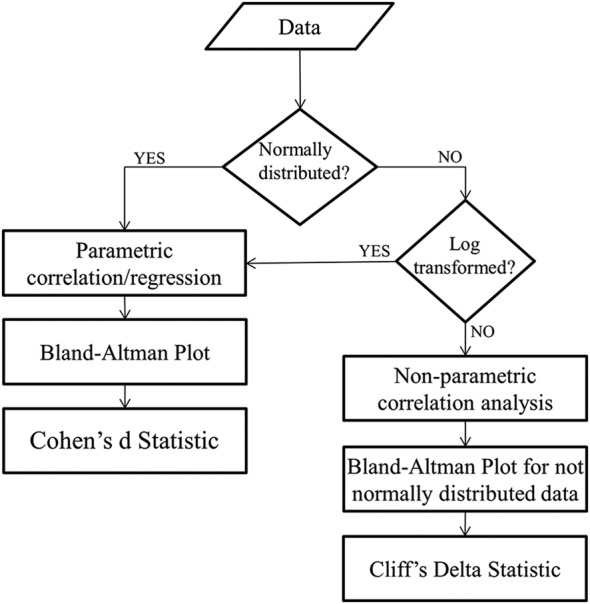
Recommendations in case of ultra-short HRV features are investigated in one condition (e.g. only at rest). All the analysis should be run between benchmark and each time scale investigated

The algorithm reported in Fig. 2 can be further articulated in the case in which the ultra-short HRV features are non-normally distributed (Fig. 3). As far as correlation tests, there are several non-parametric tests, which have been proposed. Alternatively, HRV features can be log-transformed before using a parametric test. The Bland–Altman test is parametric too, as it calculates the 95% LoA around the mean. In the case of non-normally distributed features, authors should use the same test, but investigate the dispersion around the median, and not the mean, when computing the 95% LoA. Finally, Cohen's *d* statistics assumes the normal distribution of input features, therefore it is strongly recommended to apply a log-transformation to HRV features before applying this test. Alternatively, Cliff's delta statistics should be used for non-normally distributed data as it is a non-parametric effect size measure that quantifies the amount of difference between two groups of observations beyond *p*-values interpretation [47].

**Fig. 4:**
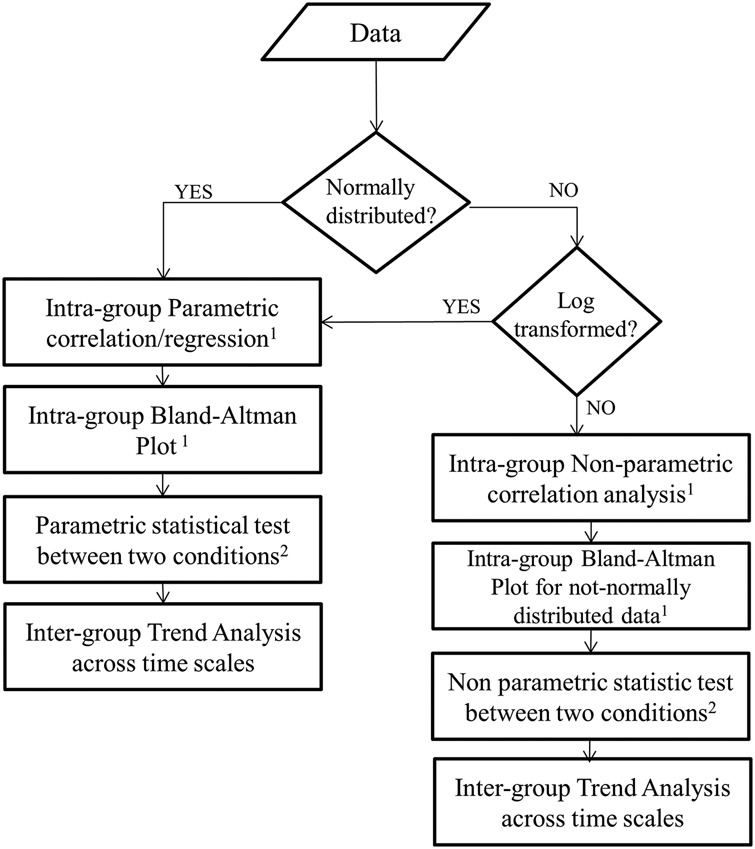
Recommendations in case of two conditions ^1^All the analysis should be run between the benchmark and each time scale investigated during both control and experimental conditions. ^2^Repeated at each time scale under investigation

In case two different conditions are explored (e.g. stress versus rest), both Figs. 2 and 3 require a further adjustment. In fact, in analogy to the best available medical practice [48], scholars should follow the algorithm proposed in Fig. 4, proving that:

- ultra-short HRV features behave as short-term ones in the same conditions (i.e. at rest or during stress), intra-group assessment;
- ultra-short HRV features maintain different behaviours in the two conditions at different lengths (i.e. if StdNN diminishes during stress, this change should be observed both at short and ultra-short term) and inter-group assessment.

As the first step, surrogate features have to be correlated with benchmark ones (i.e. short-term HRV) both in a control condition (e.g. rest phase) and during the event to be detected (e.g. stress phase). This can be verified using intra-group correlation analysis at different time lengths, i.e. in the same condition. For instance, StdNN (as well as any other HRV feature) extracted from 5 min excerpts during rest (or stress), has to be significantly correlated with StdNN extracted from any shorter 5 min excerpts during rest (or stress).

As a second step, visual investigation of bias between means (or medians for non-normally distributed features) has to be performed via Bland–Altman plots in each condition.

As the third step, the set of surrogate features has to preserve a large portion of information of the event to be detected (i.e. significance test at each time scale and/or trend analysis). This can be verified using inter-group statistical tests at each time length but in the different conditions. Therefore, scholars should verify, using a non-parametric test (unless HRV features are log transformed or normally distributed), which ultra-short HRV feature maintains statistical evidence that the median significantly differs in the two different conditions (*p* < 0.05) across time period windows. Pereira *et al.* [21] attempted to investigate which HRV features could discriminate between rest and stress using ANOVA for each selected time period window.

The fourth and last step, the trends of the HRV features (i.e. if HRV features decrease or increase during stress) should remain consistent across time lengths. In fact, a HRV feature can be assumed to maintain the same behaviour across different time lengths if the statistical significance test has *p*-value <0.05 between the control and the experimental conditions at each time scale and if the ultra-short HRV features trend consistently changes between the control and the experimental conditions with the equivalent short HRV feature (e.g., if MeanNN decreases significantly during stress at 5 min [10] this significant trend has to be consistently maintained at shorter time lengths). Once these four steps have been performed, it can be assumed that an ultra-short HRV feature is a good surrogate for the equivalent short one, if:

- the ultra-short HRV feature maintained the same behaviour between control and experimental conditions as the benchmark;
- the ultra-short HRV feature was highly and significantly correlated (e.g. correlation coefficient greater than a given threshold (e.g. 0.7) and *p*-value <0.05), with the corresponding short feature in both control and experimental conditions.

## 4 Conclusion

This review demonstrates that there is a clear lack of rigorous methods to assess the validity of ultra-short HRV features in a control situation and to identify reliable ultra-short HRV features. One of the reasons could be the lack of clear algorithm guiding scholars in proving how to identify good surrogates. Therefore, this Letter proposed, in analogy with evidence-based medicine, three algorithms, which scholars may follow to assess whether ultra-short HRV features can be considered good surrogates of short-term ones. Recommendations are given in this regard: which method should be used in each step, when intra-group or inter-group correlation and statistical tests are required, and whether those tests should be parametric or non-parametric.

## References

[1] Fenici R. Brisinda D. Sorbo A.R.: ‘Methods for real-time assessment of operational stress during realistic police tactical training’ in Kitaeff, J. (Ed.): ‘Handbook of police psychology’ (Routledge, New York, 2011), pp. 295–319

[2] Melillo P. Jovic A. De Luca N.: ‘Automatic classifier based on heart rate variability to identify fallers among hypertensive subjects’, Healthc. Technol. Lett., 2015, 2, pp. 89–94 (doi: 10.1049/htl.2015.0012) 26609412 10.1049/htl.2015.0012 PMC4612540

[3] Force T.: ‘Heart rate variability guidelines: standards of measurement, physiological interpretation, and clinical use’, Eur. Heart J., 1996, 17, pp. 354–381 (doi: 10.1093/oxfordjournals.eurheartj.a014868) 8737210

[4] Morelli D. Bartoloni L. Colombo M.: ‘Profiling the propagation of error from PPG to HRV features in a wearable physiological-monitoring device’, Healthc. Technol. Lett., 2017, pp. 1–6, doi: 10.1049/htl.2017.0039 29750114 10.1049/htl.2017.0039 PMC5933374

[5] Fleming T.R. DeMets D.L.: ‘Surrogate end points in clinical trials: are we being misled?’, Ann. Intern. Med., 1996, 125, pp. 605–613 (doi: 10.7326/0003-4819-125-7-199610010-00011) 8815760 10.7326/0003-4819-125-7-199610010-00011

[6] Gallo L. Eskicioglu C. Braga L.H.: ‘Users’ guide to the surgical literature: how to assess an article using surrogate end points’, Can. J. Surg., 2017, 60, p. 280 (doi: 10.1503/cjs.002217) 28730989 10.1503/cjs.002217 PMC5529160

[7] Yentes J.M. Hunt N. Schmid K.K.: ‘The appropriate use of approximate entropy and sample entropy with short data sets’, Ann. Biomed. Eng., 2013, 41, pp. 349–365 (doi: 10.1007/s10439-012-0668-3) 23064819 10.1007/s10439-012-0668-3 PMC6549512

[8] Massaro S. Pecchia L.: ‘Heart rate variability (HRV) analysis: a methodology for organizational neuroscience’, Org. Res. Methods, 2016, doi: 10.1177/1094428116681072

[9] Grant M.J. Booth A.: ‘A typology of reviews: an analysis of 14 review types and associated methodologies’, Health Inf. Libr. J., 2009, 26, pp. 91–108 (doi: 10.1111/j.1471-1842.2009.00848.x) 10.1111/j.1471-1842.2009.00848.x 19490148

[10] Castaldo R. Melillo P. Bracale U.: ‘Acute mental stress assessment via short term HRV analysis in healthy adults: a systematic review with meta-analysis’, Biomed. Signal Proc. Control, 2015, 18, pp. 370–377 (doi: 10.1016/j.bspc.2015.02.012)

[11] Bracale U. Melillo P. Pignata G.: ‘Which is the best laparoscopic approach for inguinal hernia repair: TEP or TAPP? A systematic review of the literature with a network meta-analysis’, Surg. Endosc., 2012, 26, (12), pp. 3355–3366 (doi: 10.1007/s00464-012-2382-5) 22707113 10.1007/s00464-012-2382-5

[12] Bracale U. Rovani M. Bracale M.: ‘Totally laparoscopic gastrectomy for gastric cancer: meta-analysis of short-term outcomes’, Minim. Invasive Ther. Allied Technol., 2012, 21, pp. 150–160 (doi: 10.3109/13645706.2011.588712) 21619505 10.3109/13645706.2011.588712

[13] Xu Q. Nwe T.L. Guan C.: ‘Cluster-based analysis for personalized stress evaluation using physiological signals’, IEEE J. Biomed. Health Inform., 2015, 19, pp. 275–281 (doi: 10.1109/JBHI.2014.2311044) 25561450 10.1109/JBHI.2014.2311044

[14] Boonnithi S. Phongsuphap S.: ‘Comparison of heart rate variability measures for mental stress detection’, Comput. Cardiol., 2011, 2011, pp. 85–88

[15] Brisinda D. Venuti A. Cataldi C.: ‘Real-time imaging of stress-induced cardiac autonomic adaptation during realistic force-on-force police scenarios’, J. Police Criminal Psychol., 2015, 30, (2), pp. 71–86 (doi: 10.1007/s11896-014-9142-5)

[16] Mayya S. Jilla V. Tiwari V.N.: ‘Continuous monitoring of stress on smartphone using heart rate variability’. 2015 IEEE 15th Int. Conf. on Bioinformatics and Bioengineering (BIBE), Belgrade, Serbia, November 2015, pp. 1–5

[17] Pandey P. Lee E.K. Pompili D.: ‘A distributed computing framework for real-time detection of stress and of its propagation in a team’, IEEE J. Biomed. Health Inform., 2016, 20, pp. 1502–1512 (doi: 10.1109/JBHI.2015.2477342) 26357414 10.1109/JBHI.2015.2477342

[18] Wijsman J. Grundlehner B. Liu H.: ‘Towards mental stress detection using wearable physiological sensors’. 2011 Annual Int. Conf. of the IEEE Engineering in Medicine and Biology Society, EMBC, Boston, MA, USA, 30 August–3 September 2011, pp. 1798–1801 10.1109/IEMBS.2011.6090512 22254677

[19] Kim D. Seo Y. Cho J.: ‘Detection of subjects with higher self-reporting stress scores using heart rate variability patterns during the day’. 30th Annual Int. Conf. of the IEEE Engineering in Medicine and Biology Society, 2008. EMBS 2008, Vancouver, BC, Canada, August 2008, pp. 682–685 10.1109/IEMBS.2008.4649244 19162747

[20] Salahuddin L. Cho J. Jeong M.G.: ‘Ultra short term analysis of heart rate variability for monitoring mental stress in mobile settings’. Conf. Proc. IEEE Eng. Medicine Biology Society, Lyon, France, August 2007, pp. 4656–4659 10.1109/IEMBS.2007.4353378 18003044

[21] Pereira T. Almeida P.R. Cunha J.P.: ‘Heart rate variability metrics for fine-grained stress level assessment’, Comput. Methods Programs Biomed., 2017, 148, pp. 71–80 (doi: 10.1016/j.cmpb.2017.06.018) 28774440 10.1016/j.cmpb.2017.06.018

[22] Papousek I. Nauschnegg K. Paechter M.: ‘Trait and state positive affect and cardiovascular recovery from experimental academic stress’, Biol. Psychol., 2010, 83, pp. 108–115 (doi: 10.1016/j.biopsycho.2009.11.008) 19944130 10.1016/j.biopsycho.2009.11.008

[23] Sun F.-T. Kuo C. Cheng H.-T.: ‘Activity-aware mental stress detection using physiological sensors’. Int. Conf. on Mobile Computing, Applications, and Services, Santa Clara, CA, USA, October 2010, pp. 211–230

[24] Schubert C. Lambertz M. Nelesen R.: ‘Effects of stress on heart rate complexity—a comparison between short-term and chronic stress’, Biol. Psychol., 2009, 80, pp. 325–332 (doi: 10.1016/j.biopsycho.2008.11.005) 19100813 10.1016/j.biopsycho.2008.11.005 PMC2653595

[25] De Rivecourt M. Kuperus M. Post W.: ‘Cardiovascular and eye activity measures as indices for momentary changes in mental effort during simulated flight’, Ergonomics, 2008, 51, pp. 1295–1319 (doi: 10.1080/00140130802120267) 18802817 10.1080/00140130802120267

[26] Choi J. Gutierrez-Osuna R.: ‘Using heart rate monitors to detect mental stress’. Sixth Int. Workshop on Wearable and Implantable Body Sensor Networks, 2009. BSN 2009, Berkeley, CA, USA, June 2009, pp. 219–223

[27] Hjortskov N. Rissén D. Blangsted A.K.: ‘The effect of mental stress on heart rate variability and blood pressure during computer work’, Eur. J. Appl. Physiol., 2004, 92, pp. 84–89 (doi: 10.1007/s00421-004-1055-z) 14991326 10.1007/s00421-004-1055-z

[28] Wang X. Ding X. Su S.: ‘Genetic influences on heart rate variability at rest and during stress’, Psychophysiology, 2009, 46, pp. 458–465 (doi: 10.1111/j.1469-8986.2009.00793.x) 19226307 10.1111/j.1469-8986.2009.00793.x PMC3713464

[29] Li Z. Snieder H. Su S.: ‘A longitudinal study in youth of heart rate variability at rest and in response to stress’, Int. J. Psychophysiol., 2009, 73, pp. 212–217 (doi: 10.1016/j.ijpsycho.2009.03.002) 19285108 10.1016/j.ijpsycho.2009.03.002 PMC2719684

[30] Esco M.R. Flatt A.A.: ‘Ultra-short-term heart rate variability indexes at rest and post-exercise in athletes: evaluating the agreement with accepted recommendations’, J. Sports Sci. Med., 2014, 13, pp. 535–541 25177179 PMC4126289

[31] Nardelli M. Greco A. Bolea J.: ‘Reliability of Lagged Poincaré Plot parameters in ultra-short heart rate variability series: application on affective sounds’, IEEE J. Biomed. Health Inform., 2017, PP, (99), pp. 1–1, (doi: 10.1109/JBHI.2017.2694999) 10.1109/JBHI.2017.2694999 28436907

[32] Thong T. Li K. McNames J.: ‘Accuracy of ultra-short heart rate variability measures’. Proc. 25th Annual Int. Conf. of the IEEE Engineering in Medicine and Biology Society, 2003, Cancun, Mexico, September 2003, pp. 2424–2427

[33] Munoz M.L. van Roon A. Riese H.: ‘Validity of (ultra-) short recordings for heart rate variability measurements’, PLoS ONE, 2015, 10, p e0138921 (doi: 10.1371/journal.pone.0138921) 26414314 10.1371/journal.pone.0138921 PMC4586373

[34] Flatt A.A. Esco M.R.: ‘Validity of the ithlete™ smart phone application for determining ultra-short-term heart rate variability’, J. Hum. Kinet., 2013, 39, pp. 85–92 (doi: 10.2478/hukin-2013-0071) 24511344 10.2478/hukin-2013-0071 PMC3916914

[35] Nussinovitch U. Elishkevitz K.P. Katz K.: ‘Reliability of ultra-short ECG indices for heart rate variability’, Ann. Noninvasive Electrocardiol., 2011, 16, pp. 117–122 (doi: 10.1111/j.1542-474X.2011.00417.x) 21496161 10.1111/j.1542-474X.2011.00417.x PMC6932379

[36] McNames J. Aboy M.: ‘Reliability and accuracy of heart rate variability metrics versus ECG segment duration’, Med. Biol. Eng. Comput., 2006, 44, pp. 747–756 (doi: 10.1007/s11517-006-0097-2) 16960742 10.1007/s11517-006-0097-2

[37] Baek H.J. Cho C.-H. Cho J.: ‘Reliability of ultra-short-term analysis as a surrogate of standard 5-min analysis of heart rate variability’, Telemed. E-Health, 2015, 21, pp. 404–414 (doi: 10.1089/tmj.2014.0104) 10.1089/tmj.2014.0104 25807067

[38] Schroeder E.B. Whitsel E.A. Evans G.W.: ‘Repeatability of heart rate variability measures’, J. Electrocardiol., 2004, 37, pp. 163–172 (doi: 10.1016/j.jelectrocard.2004.04.004) 15286929 10.1016/j.jelectrocard.2004.04.004

[39] Kwon S. Lee D. Kim J.: ‘Sinabro: a smartphone-integrated opportunistic electrocardiogram monitoring system’, Sensors, 2016, 16, p. 361 (doi: 10.3390/s16030361) 10.3390/s16030361 PMC4813936 26978364

[40] Arza A. Garzón J. Hemando A.: ‘Towards an objective measurement of emotional stress: preliminary analysis based on heart rate variability’. 2015 37th Annual Int. Conf. of the IEEE Engineering in Medicine and Biology Society (EMBC), Milan, Italy, August 2015, pp. 3331–3334 10.1109/EMBC.2015.7319105 26737005

[41] Salahuddin L. Jeong M.G. Kim D.: ‘Ultra short term analysis of heart rate variability using normal sinus rhythm and atrial fibrillation ECG data’. 2007 9th Int. Conf. on e-Health Networking, Application and Services, 2007, pp. 240–243

[42] Rice W.R.: ‘Analyzing tables of statistical tests’, Evolution, 1989, 43, pp. 223–225 (doi: 10.1111/j.1558-5646.1989.tb04220.x) 28568501 10.1111/j.1558-5646.1989.tb04220.x

[43] Benjamini Y. Hochberg Y.: ‘Controlling the false discovery rate: a practical and powerful approach to multiple testing’, J. R. Stat. Soc. B, Methodol., 1995, 57, (1), pp. 289–300

[44] Vidakovic B.: ‘Statistics for bioengineering sciences: with MATLAB and WinBUGS support’ (Springer Science & Business Media, New York, NY, USA, 2011)

[45] Dewitte K. Fierens C. Stöckl D.: ‘Application of the Bland–Altman plot for interpretation of method-comparison studies: a critical investigation of its practice’, Clin. Chem., 2002, 48, pp. 799–801 11978620

[46] Watson P. Petrie A.: ‘Method agreement analysis: a review of correct methodology’, Theriogenology, 2010, 73, pp. 1167–1179 (doi: 10.1016/j.theriogenology.2010.01.003) 20138353 10.1016/j.theriogenology.2010.01.003

[47] Macbeth G. Razumiejczyk E. Ledesma R.D.: ‘Cliff's delta calculator: a non-parametric effect size program for two groups of observations’, Universitas Psychol., 2011, 10, pp. 545–555

[48] Li Z. Chines A. Meredith M.: ‘Statistical validation of surrogate endpoints: is bone density a valid surrogate for fracture?’, J. Musculoskeletal Neuronal Interact., 2004, 4, p. 64 15615079

